# Cortisol as a Stress Indicator in Fish: Sampling Methods, Analytical Techniques, and Organic Pollutant Exposure Assessments

**DOI:** 10.3390/ijerph20136237

**Published:** 2023-06-27

**Authors:** Leila Soledade Lemos, Leo Moreira Angarica, Rachel Ann Hauser-Davis, Natalia Quinete

**Affiliations:** 1 Institute of Environment, Florida International University, North Miami, FL 33181, USA; 2 Department of Chemistry and Biochemistry, Florida International University, North Miami, FL 33181, USA; 3 Laboratório de Avaliação e Promoção da Saúde Ambiental, Instituto Oswaldo Cruz, Fundação Oswaldo Cruz (Fiocruz), Rio de Janeiro 21040-900, RJ, Brazil

**Keywords:** glucocorticoid, immunoassays, LC-MS/MS, matrix sampling methods, stress responses, teleost

## Abstract

Cortisol is the main glucocorticoid released during stress responses in most fish and has been employed to investigate different stressors, including organic pollutants. This review discusses shifts in cortisol concentrations and examines different matrix sampling methods (invasive vs. minimally or non-invasive) and the main analytical cortisol determination techniques (immunoassays and liquid chromatography-tandem mass spectrometry). Assessments on organic pollutant exposure in fish and associated adverse effects are also discussed. Studies in this regard may aid in identifying organic pollutant toxicological modes of action, mechanistic response, toxicokinetics, and toxicodynamics, as well as pollution sources and associated health risks in fish, ultimately aiding in the development of effective management strategies to mitigate the impacts of organic pollutants on fish populations and their associated ecosystems.

## 1. Introduction

Stressors are often categorized as harmful when leading to organism death [1,2,3]. However, this is a very extreme criterion where preventive measures are not possible. Thus, sublethal biological endpoints comprising detectable biochemical and tissue-level changes leading to molecular, biochemical, and physiological alterations, termed biomarkers, should be employed to evaluate stressor effects [4]. The biomarker approach is, therefore, paramount in identifying early responses to different stressors, allowing for stressor mechanisms of action and potential ecological effect determinations [5]. Additionally, this approach is more sensitive in identifying individual physiological responses.

Many aquatic organisms are recognized bioindicators for different types of stressors, including both biotic (e.g., disease and parasites) and abiotic (e.g., temperature, chemical contamination, photoperiod, and salinity) factors [6,7,8,9]. Furthermore, organismal behavioral, physiological, and biochemical responses are highly relevant and particularly useful in different stressor evaluations. Fish are more sensitive to stressors than many other vertebrates [10], making them ideal candidates for stress response assessments in different fields, such as aquaculture, ecotoxicology, ecology, endocrinology, human health risks, and public health assessments.

Stress responses in fish initiate at the molecular and cellular levels. When left unchecked, these responses may disturb the individual homeostatic state and lead to physiological and metabolic alterations and potentially lead to organismal functional impairment [11]. Primary responses include endocrinological changes, such as increased catecholamine and corticosteroid concentrations. Secondary responses include metabolic changes such as hydromineral balance and cardiovascular, respiratory, and immune functions (e.g., increased glucose and lactate levels, increased heat-shock protein production, and antibody production alterations). Finally, tertiary responses include changes in whole-animal performance (e.g., growth, disease resistance, and modified behavioral patterns, [12]). Among different stress response biomarkers, cortisol has been shown to be a reliable stress indicator towards organic pollutants in fish (e.g., [13]), with practical applications in biomonitoring efforts.

To date, several methods have been used for cortisol determination in fish, including different types of matrices and varied field and analytical techniques. Gaining a thorough comprehension of the characteristics of different types of matrices and their sampling methods and analysis is crucial. This includes comprehending the invasive nature of the sampling process, the degree of accuracy involved, the representation of plasma concentrations, the reflection of acute or chronic stress levels, and the assessment of variability among samples. Analytical techniques employed in this regard encompass a range of different methodologies, including enzyme-linked immunosorbent assay (ELISA), radioimmunoassay (RIA), and liquid chromatography-tandem mass spectrometry (LC-MS/MS). Each method has its own strengths and limitations, and selecting the appropriate technique depends on factors such as sensitivity, specificity, cost-effectiveness, and feasibility in field or laboratory settings. The choice of appropriate matrix and techniques will ensure minimized harm to the study object and a reliable cortisol concentration measurement, enabling researchers to draw meaningful conclusions from their findings. All these topics will be further examined in subsequent sections of this review.

Thus, this review aims to provide a comprehensive resource of the various sampling and analytical techniques employed for the determination of cortisol in fish, including the different matrices, techniques, and the advantages and disadvantages of each method. This review is more comprehensive than previous reviews, building upon and going beyond a previous seminal review [14], reporting and discussing recent contributions to the literature, including cortisol correlations between different fish matrices (e.g., plasma, muscle, feces, skin mucus and gut content), and new extraction techniques (i.e., high-throughput thin-film solid-phase microextraction) allowing for cortisol determination in small sample amounts by LC-MS/MS. Furthermore, this review also aims to provide a compilation of organic pollutant exposure assessments in fish along with the corresponding physiological response indicated by changes in cortisol concentrations. A better understanding of the mechanistic action and physiological responses exerted in fish by these pollutants can aid in identifying pollution sources, implementing targeted remediation strategies, and developing robust regulatory measures, ensuring the well-being of fish populations.

## 2. Methods

The present study employed a narrative or traditional literature review methodology to comprehensively overview the existing literature on the available sampling methods, matrices, and analytical techniques to determine cortisol in fish. Additionally, we examined the literature on organic pollutant exposure assessments in fish along with the corresponding cortisol responses. To identify relevant studies, searches were conducted in different electronic databases, namely Google Scholar and PubMed, combining keywords related to the research topic, such as [cortisol, fish, matrices], [cortisol, fish, sampling methods], [cortisol, fish, analytical techniques], and [cortisol, fish, organic pollutants]. The search was not limited to a specific language or time frame.

## 3. Cortisol in Fish

Cortisol is a reliable stress indicator produced by the adrenal cortex in the adrenal glands in vertebrates and is considered the major stress-related hormone in teleost fish [14]. This glucocorticoid (GC) hormone has been widely employed to evaluate several stressor effects in fish, from disease to chemical contaminants [15,16,17], water temperature, and photoperiod [18,19,20,21,22], among others.

While cortisol is typically associated with negative stressors, it is important to acknowledge that not all cortisol fluctuations in fish are indicative of distress. This may include changes in cortisol concentrations related to environmental cycles, circadian rhythms, sex, maturity, and reproductive stages [21,22,23,24]. Some changes in cortisol concentrations can also be attributed to adaptive behaviors or eustress, which are stimuli that elicit a positive response, such as becoming alert and engaging in activities like foraging and breeding [1,3]. Thus, it is of extreme importance to differentiate baseline (a normal range within a healthy population; homeostasis state is not impaired) from stress-related concentrations (outside of the normal range; [1,25]) accounting for typical cortisol variations within an organism, including environmental cycles, circadian rhythms, sex, maturity, and reproductive stages [21,23,24].

The mechanistic action of cortisol starts with the release of the polypeptide corticotropin-releasing hormone (CRH) into the blood by the hypothalamus. CRH further stimulates the secretion of the adrenocorticotrophic hormone (ACTH) from the anterior pituitary gland. ACTH then activates the melanocortin 2 receptor (MC2-R) in the adrenal cortex to initiate the synthesis and release of cortisol and other GCs by the inter-renal tissue [26,27]. This complex neuroendocrine system forms the HPA axis (hypothalamic-pituitary-adrenal axis).

The only physiologically active form of cortisol consists of unbound molecules found in plasma, which are cleared by molecule breakdown, resulting in cortisone release, or by tissue uptake [14]. Clearance rates may vary on the type of stressor (i.e., acute or chronic, [28]), returning to basal levels at varying post-stress times [29]. Tissue uptake likely occurs through passive diffusion since cortisol molecules are hydrophobic and highly lipophilic. In tissues, cortisol molecules bind to GC and mineralocorticoid hormone receptors to modulate gene expression [30] and other non-genomic actions that might be relatively independent of specific cortisol receptors [31]. Cortisol molecules are then metabolized or inactivated, and the produced metabolites (e.g., cortisone) are released into the environment through urine and feces, but mainly via the liver-bile-feces route. The free form of cortisol can also diffuse through the gills [32] and be released into the surrounding water.

## 4. Invasive vs. Non-Invasive Biological Sampling Methods for Cortisol Determinations

**Figure 1:**
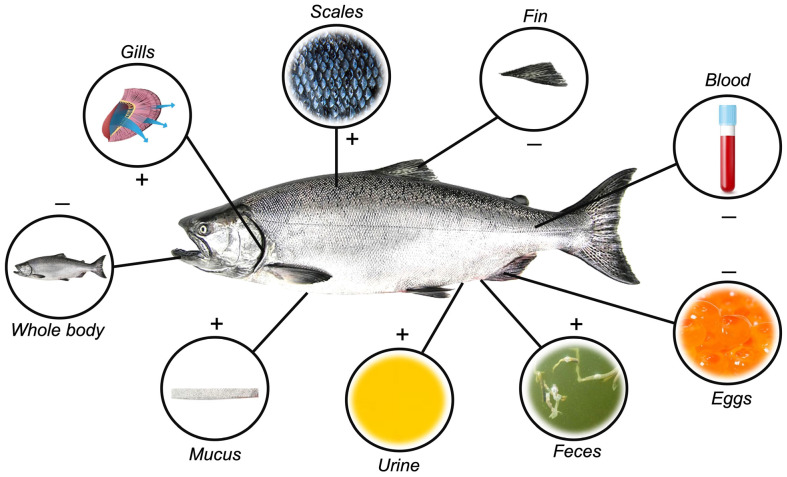
Different matrices are used to quantify stress-response hormones in fish. Positive signs indicate non-invasive matrix removal obtainment, while negative signals indicate invasiveness.

Initially, samples employed in stress-related biomarker evaluations were obtained almost exclusively through invasive methods, restricted mostly to whole blood (which can be processed into serum or plasma) and internal organs and tissues, such as liver, kidney, gills, gonads, eggs, and whole body [33,34,35,36] (Figure 1). Technological advances have, however, continuously evolved towards minimally invasive to non-invasive practices, which now may include the use of surrounding water, urine, skin mucus, scales, and fecal samples [32,37,38,39,40,41,42,43,44] (Figure 1). Several advantages and disadvantages are associated with each sample matrix, depicted in Table 1.

Methods employed to assess the whole body, gonads, and eggs are all lethal, not allowing for repeated measurements of the same individuals. On the other hand, these matrices guarantee high accuracy concerning individual cortisol concentrations, except for fish with small eggs that must be pooled to guarantee a minimum mass for analysis [14]. Furthermore, whole-body concentrations have been verified as being highly correlated to plasma [45,46].

Plasma, fins, and scales are routinely used to quantify cortisol at the individual level, although direct contact with the animal to be sampled is required, which could potentially alter stress response levels, increasing cortisol concentrations and leading to biased basal estimates [14]. Plasma and fin assessments guarantee high measurement accuracy and can be used to quantify both basal and stress response levels, while scales indicate chronic stress, making it hard to associate the response to a specific stimulus.

Feces, mucus, and water are obtained non-invasively, in which cortisol concentrations reflect plasma concentrations [47,48]. All three are used to quantify cortisol concentrations at the individual level, except for surrounding water samples in tanks that house more than one individual. Water samples, however, are also considered highly accurate and representative of circulating plasma concentrations [47,48]. Another advantage of using mucus is that it is well associated with the timing of the stimulus, being able to associate cause and response. In contrast, a lag time between stimulus and excretion is noted for fecal cortisol, directly correlated with gut transit time, which can take from less than 30 min to over 24 h, depending on the species and individual [49]. Another disadvantage of using fecal samples is that relatively higher sample sizes are required, as low masses can lead to inflated results [50,51].

Regarding inter-sample variability, lower variations are noted for plasma, eggs, and feces, while water sample variability is considered moderate, and skin mucus is high [14].

Fish stress response assessments are shifting away from the use of invasive samplings (e.g., blood) to samples alternatively obtained by non-invasive or minimally invasive methods, as many have shown to faithfully reflect circulating cortisol concentrations in stressor-exposed fish [52]. This also allows for lower individual disturbances and repeated measurements over time [14].

For example, Ding et al. [39] compared plasma cortisol concentrations to feces in Atlantic salmon (*Salmo salar*) using the ELISA method. These authors reported strong positive correlations between both matrices, indicating that feces concentrations reflect plasma changes and can be used as an alternative non-invasive matrix. The authors also reported that both plasma and fecal cortisol and metabolite concentrations increased within 40 min after the applied stimulus in small fish (<100 g). In another study, Uren Webster et al. [43] compared cortisol concentrations in feces, skin mucus, and plasma in Atlantic salmon fry, also by ELISA. All matrices exhibited positive correlations with each other, although the plasma-mucus correlation was observed to a lesser extent. The authors indicated that elevated fecal cortisol concentrations were associated with pronounced changes in fecal microbiome diversity and structure, while cortisol concentrations in skin mucus were not associated with changes in the skin microbiome. Simontacchi et al. [53] also quantified cortisol concentrations in different sea bass matrices (plasma, skin mucus, gut contents, and muscle) under different conditions (handling, stocking density, time spent in confinement tanks, and ice treatment) to create different stress conditions. Their results verified higher concentrations in plasma (679 ng·mL^−1^), followed by muscle (198 ng·mL^−1^), skin mucus (70.40 ng·mL^−1^), and gut content (57.10 ng·mL^−1^). In another assessment conducted by Bertotto et al. [54], the authors assessed cortisol concentrations in different matrices of three different fish species. Higher concentrations were observed in plasma, followed by muscle, skin mucus, and gut content in sea bass, while higher concentrations were noted in plasma, followed by muscle, fins, and skin mucus in common carp. Finally, in rainbow trout, higher concentrations were found in plasma, followed by fins, gut content, muscle, and skin mucus, demonstrating inter-species variations. Also recently, Gozdowska et al. [55] used skin samples of three-spined sticklebacks (*Gasterosteus aculeatus*) from the southern Baltic Sea to simultaneously investigate if melatonin, AFMK (N1-acetyl-N2-formyl-5-methoxykynuramine; biologically active metabolite of melatonin), and cortisol would create a local stress response system in fish, similarly to what is known to occur in mammals. Oxidative stress in the individuals was then induced by exposing them to potassium dichromate. The discussion involved the likelihood of this fact being related to a strategy to cope with oxidative stress, where both components act locally to prevent damage caused by active oxygen molecules. This study demonstrates the value of a more holistic approach, simultaneously evaluating different biomarkers to reach a better understanding of how different physiological systems interact and respond to stressors, shedding light on a potential adaptive strategy employed by fish to cope with oxidative stress.

Multiple studies have also reported positive correlations between mucus and plasma cortisol concentrations [53,54,55,56]. When assessing cortisol concentrations in plasma, mucus, gut contents, and muscle of sea bass (*Dicentrarchus labrax*) before and after stressful situations, Simontacchi et al. [53] reported clear and significant correlations between plasma and the other evaluated matrices, with concentrations 10–30-fold higher concentrations detected in plasma. However, the profiles of all four matrices were similar, indicating inter-renal gland production. The authors also investigated potential correlations between plasma cortisol and glucose and lactate levels, with no correlations detected, indicating that these variables are not reliable stress response indicators in fish. Another study compared cortisol concentrations in plasma, mucus, and scales in juvenile rainbow trout (*Oncorhynchus mykiss* [57]), observing that both mucus and scales reflect circulating cortisol concentrations, but that concentrations are strongly correlated with chronic stress in scales, indicating that scales likely provide retrospective measurements of previous stress responses.

Cortisol concentrations in eggs have also been correlated to maternal circulating concentrations just before spawning [35,58]. Stratholt et al. [35], for example, exposed a group of adult coho salmon females (*Oncorhynchus kisutch*) during late oogenesis to physical stress and detected elevated plasma and egg cortisol concentrations compared to a control group. Bertotto et al. [54] used RIAs to compare cortisol concentrations in plasma, skin mucus, gut contents, lateral muscle, and caudal fin of sea bass, common carp (*Cyprinus carpio*), and rainbow trout before and after stressful conditions. Sea bass cortisol concentrations increased significantly in plasma (2.5-fold), mucus (4-fold), and muscle (9-fold), demonstrating that these matrices are valuable in acute stress assessments. In common carp, cortisol concentrations increased in plasma, mucus, muscle, and fins, with the latter presenting almost the same increases as muscle, while rainbow trout concentrations increased the most in all matrices, from 12 to 29-fold.

Therefore, it is clear that cortisol concentrations in many of the matrices are highly correlated with circulating plasma concentrations and can be used as alternative endocrinological tools to assess stress. However, it is important to note that they may indicate different types of stress, namely acute (e.g., plasma, mucus, muscle) or chronic (e.g., scales). Furthermore, concentrations may widely vary according to species and individual.

Finally, a very useful non-invasive tool comprises measuring cortisol in the surrounding water, made possible due to the presence of multiple excretion pathways from fish to water, including via gills, urine, feces, skin, and ovarian fluid. Previous studies have reported that the gills are the main source of free steroids in water [32] and although concentrations are normally very low in the water, they may be quantified following pre-concentration techniques through the use of liquid–liquid extraction employing organic solvents, such as diethyl ether, or solid phase extraction cartridges containing octadecylsilane.

In a literature review on associations between steroid concentrations (17,20β (dihydroxy)-P (pregen) and T (trihydroxy)) in water and in plasma, Scott and Ellis [32] concluded that free steroids found in water are a result of gill leakage due to the concentration gradient between plasma and water. The authors argued that free steroid concentrations in water equate to the concentration of physiologically active steroids in plasma. They also highlighted various advantages of measuring steroids in water, mainly that the procedure requires minimal interventions, such as bleeding or exposure to a stressor. Other advantages include unbiased results, the ability to repeat measurements on the same fish, perform safe measurements on very small specimens, allowing for assessing integrated responses of numerous fish, and the ability to monitor behavioral and physiological factors. On the other hand, challenges include water concentrations mainly influenced by plasma concentrations and steroid affinity for specific binding proteins in blood, as well as blood flow rates in gills, among others.

## 5. Main Analytical Methods Employed in Cortisol Quantitation

Several methods are available to quantify cortisol and its metabolites directly from biological samples [40]. In this regard, RIAs were very popular, being, however, gradually replaced by ELISAs due to technological advancement and attempts to reduce laboratory waste and exposure to toxic substances [14].

Immunoassays are very sensitive methods, with limits of detection (LOD) as low as the pg·mL^−1^ range [59,60]. This is due to indirect, immunoglobin-based measurement technologies that bind the target compound and amplify the signal through a radioactive signal, as in RIA, or an enzymatic change, as in ELISA [60,61]. Despite their high sensitivity, cross-reactivity may occur in these assays when the assay antibody binds to endogenous molecules displaying similar structures to the target analyte [40]. This is especially true for matrices such as urine and feces, in which metabolites are more abundant than the target analyte [62]. Thus, the amplification technologies that allow high immunoassays’ sensitivity are often less reliable than direct quantification methods [63]. Another major limitation associated with immunoassays comprises the fact that these techniques are designed for the specific measurement of single hormones per assay, limiting the ability to determine multiple hormones per sample and increasing costs due to the need to buy multiple assay kits.

**Figure 2:**
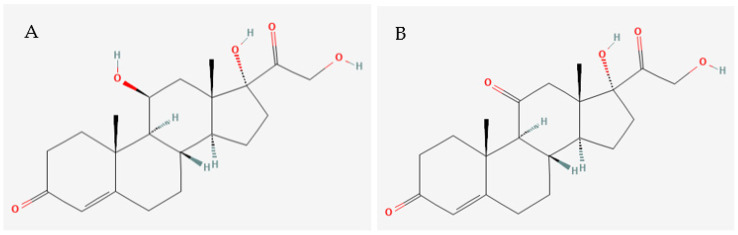
Chemical structures of (**A**) cortisol and (**B**) cortisone. Source: Pubchem (2023).

Several studies have highlighted the need for more sensitive and specific analytical tools able to determine the concentrations of structurally similar molecules, such as cortisol and cortisone [64,65] (Figure 2).

In this regard, LC-MS/MS has become one of the most common techniques available in laboratories applied to this end. This technique, coupled with Electrospray Ionization (ESI), which uses a high voltage electrospray to produce ions from liquid samples, is currently one of the most commonly applied techniques for hormone determinations employing LC-MS or LC-MS/MS [40]. Overall, LC-MS/MS provides a robust platform with sufficient sensitivity and specificity needed for measuring steroid hormones [40], integrating the physical separation capabilities of liquid chromatography with the mass analysis capabilities of mass spectrometry [66]. Furthermore, very small sample amounts are required in LC-MS/MS assessments, a high-throughput and high-specificity method, able to detect several hormones compared to other methods such as immunoassays.

In this regard, Nouri et al. [67] showcased the versatility of the LC-MS/MS technique by determining 14 different steroid hormones, including cortisol, in low-volume serum (10 μL) and tissue homogenates of fish. The use of low-volume serum increased the detection limit to 0.012 ng·mL^−1^, while a higher serum volume (245 μL) improved the LOD 2 to 40-fold. The method was also compared with RIA for two of the steroid hormones (testosterone and 17β-estradiol), displaying a high correlation between both methods. The method was then validated for four of the hormones in low and high plasma volumes of largemouth bass (*Micropterus salmoides*). The largemouth bass females displayed higher mean cortisol concentrations (low volume: 43.50 ng·mL^−1^; high volume: 41.10 ng·mL^−1^) than the males (low volume: 104.30 ng·mL^−1^; high volume: 91.60 ng·mL^−1^). They also developed a method and quantified hormones in whole-body fish homogenates of small fish (fathead minnow—*Pimephales promelas*; zebrafish—*Danio rerio*; and silverside—*Menidia beryllina*) and compared the values to plasma concentrations, finding great consistency between the two matrices, suggesting that whole tissue homogenates are reliable alternatives for hormone quantification when plasma volume is not sufficient.

In another study, Maciążek-Jurczyk et al. [68] developed an in vitro high-throughput thin-film solid-phase microextraction (TF-SPME)-LC-MS/MS method for targeted analysis of varied hormones (i.e., cortisol, testosterone, progesterone, estrone (E1), 17β-estradiol (E2), and 17α-ethinylestradiol (EE2)) in low concentrated plasma of wild white sucker (*Catostomus commersonii*). This method allowed the direct extraction and simultaneous determination of free and total hormone concentrations without prior sample preparation or dilution. Free cortisol concentrations ranged from 13.26 to 20.87 ng·mL^−1^, while total cortisol concentrations ranged from 16.15 to 21.50 ng·mL^−1^ in female and male white suckers, respectively. The novel technique demonstrates an advance in the field, allowing for repeated sampling and investigation of the influence of factors on the studied subjects.

In a more recent study, Meling et al. [42] developed a novel technique for measuring fecal cortisol in Atlantic salmon (*Salmo salar*) from Norway, comprising an enzymatic hydrolysis, liquid–liquid extraction, derivatization, and analysis by LC-MS/MS. Cortisol concentrations were relatively high in fish released into seawater, with a concentration of 437 ng·g^−1^ four days after the transfer, but were significantly decreased to 74 ng·g^−1^ after eight days of the transfer.

The main challenge of the LC-MS/MS technique, however, is the fact that several biological molecules, such as steroids, present similar chemical structures and fragmentation patterns, making their separation complicated, especially for isomers with identical masses [63]. Therefore, clear separation and unique fragmentation patterns are necessary for accurate results and biological interpretations. For example, water loss is a common fragmentation pattern not specific to hormones and may lead to misidentification in complex biological matrices where background ion detection may affect the target measurement [63]. Furthermore, some studies have emphasized that the detection of several hormones in very low volumes of serum or plasma is still challenging (e.g., [67]). Furthermore, LC-MS/MS has improved specificity and higher throughput, being a faster technique when measuring several hormones compared to other methods, such as immunoassays.

Some studies have also employed gas chromatography-tandem mass spectrometry (GC-MS) to quantify steroid hormones. However, this method requires chemical derivatization to increase target compound volatility and reduce the thermal instability and polarity of steroid hormones, increasing workflow steps [63,69,70,71,72]. Furthermore, although gas chromatography offers multi-class profiling potential, excellent chromatographic resolutions, and ready characterization of molecule structures by intensive fragmentation, a number of pitfalls are noted concerning steroid quantification [40], mainly concerning reproducibility due to incomplete derivatization. Consequently, this technique is not as applied as LC-MS.

Table 2 below summarizes the advantages and disadvantages of each of these techniques, which should be considered when assessing cortisol in fish.

## 6. Cortisol in Fish as a Response towards Organic Pollutants

Several studies have reported cortisol as a stress response towards organic pollutants from different classes in exposed fish e.g., [73,74,75,76]. This is not surprising, as many organic pollutants act as endocrine disruptors, with direct effects on adrenal development and steroidogenesis [77], and may affect multiple biological systems, including cholesterol biosynthesis, cardiac and hematological function, peripheral and central nervous system function, stress response, and osmoregulatory and acid-base balance processes [78,79].

Pollutants have been postulated to affect fish stress responses by acting as stressors, activating the HPA axis, where high short-term cortisol concentrations provide protection against toxic effects [80,81]. They may also directly affect the functioning of target cells, such as inter-renal cells, for example, impairing protein and enzyme functions, leading to oxidative imbalances due to increased reactive oxygen species production, impairing cortisol production through oxidative stress mechanisms [82].

Prolonged high cortisol concentrations, however, may result in several deleterious effects, including immunosuppression and reduced reproductive investment in sex steroid production (see [83] for further details). HPA axis exhaustion has also been noted in this context, eventually leading to negative feedback on the axis, down-regulating several receptors and leading to cell atrophy (see [83] for further details).

It is also important to note that female stress response when carrying eggs may also directly affect developing embryos, as several hormones, such as cortisol, are transferred to the developing progeny by the mother, which may result in immunosuppression, while females affected in the opposite manner, by decreasing cortisol concentrations during stress, may lead to reduced embryo immunocompetence [84].

In addition, many fish inhabit highly contaminated areas, suffering chronic exposure to complex contaminant mixtures within a multi-stressor framework. This, therefore, comprises a significant challenge concerning stress responses in aquatic environments; organisms are exposed to complex contaminant mixtures within a multi-stressor framework [85]. This, in turn, leads to interactive effects (i.e., synergistic, antagonistic, and additive). In this regard, some regulatory agencies (i.e., in Europe) have “incorporated the combined effects of chemical contaminants, different experimental approaches for providing combined effect data, the involvement of biomarkers to characterize modes and mechanisms of action and toxicity pathways, and efforts to identify relevant risk scenarios related to combined effects” in their ongoing environmental risk assessment guidelines and manual developments, as stated by Dietz et al. [85]. Some studies have, therefore, indicated that major physiological stress response traits should, therefore, be determined in these scenarios, including hormones such as cortisol, associated with metabolic balance indicators (i.e., metabolites, cellular energy allocation, energy reserves, and global condition indices) and, finally, cellular defenses [86]. Assessments applying this type of approach, however, are still limited.

Concerning field assessments, Hontela et al. [74], for example, demonstrated that yellow perch (*Perca flavescens*) and northern pike (*Esox lucius*) from sites highly polluted by polycyclic aromatic hydrocarbons (PAHs) and polychlorinated biphenyls (PCBs) exhibited cortisol increases with increasing pollution levels, presenting elevated serum cortisol as well as large pituitary corticotropes, with plasma cortisol concentrations increasing to about 10 ng·mL^−1^ compared to their basal range (0–5 ng·mL^−1^, [33]). The authors postulated that lifelong exposure to such pollutants might lead to the exhaustion of the cortisol-producing endocrine system, probably due to prolonged system hyperactivity, potentially leading to long-term physiological impairments. In another study, Pottinger and colleagues [87] reported increased cortisol concentrations in three-spined sticklebacks (*Gasterosteus aculeatus*) sampled at downstream sites of rivers characterized by relatively high nitrate, nitrite, and ammonia concentrations in NW England compared to upstream sites, indicating potential disruption on the neuroendocrine stress axis function in the sampled fish. In contrast, one study [83] assessed cortisol concentrations in round goby (*Neogobius melanostomus*) sampled from well-known PAH and metal-contaminated areas and reported similar cortisol and lactate levels following a stress event for fish from both areas. The authors indicated that not all fishes demonstrated impaired stress responses, even in highly contaminated habitats. They also indicated that such animals may still display increased vulnerability to predation, as fish from the contaminated site tired twice more rapidly than fish from the cleaner site, probably due to contamination effects.

Several laboratory fish exposure assessments have also been conducted, both in vivo and in vitro, to assess stress responses to organic pollutants from several classes commonly detected in aquatic systems, such as per- and polyfluoroalkyl substances (PFAS), PCBs, PAHs, and phthalates. Concerning PFAS, for example, Mortensen et al. [88] fed juvenile Atlantic salmon (*Salmo salar*) gelatin capsules containing fish food spiked with PFOA or PFOS (0.2 mg·kg^−1^ fish) and methanol. PFOA and PFOS exposures led to significant decreases in plasma cortisol concentrations, as well as estrone and testosterone, while 17α-methyltestosterone and cholesterol significantly decreased with PFOA exposure and increased with PFOS exposure on sampling day 2. PFOA, on the other hand, significantly increased estrone and testosterone, and no effects were observed for cortisol, 17α-methyltestosterone, and cholesterol at sampling day 5. PFOS and PFOA produced an apparent time-dependent increase in kidney CYP3A, CYP1A1, and glutathione-S-transferase (GST) expression, while similar effects were only temporary in the liver. The changes in plasma steroid hormone concentrations paralleled changes in CYP3A mRNA levels, shedding light on mechanistic PFAS’ mode of action.

Regarding phthalates, Park et al. [89] assessed the effects of mono-(2-ethylhexyl) phthalate (MEHP), one of the major phthalate metabolites, on the endocrine activity in adult male and female zebrafish as a proxy for reproductive dysfunction. Female zebrafish exposed to 50 μg·mL^−1^ MEHP for 21 days produced fewer ovulated eggs as well as the hepatic VTG mRNA abundance compared to a control group, while higher 17β-estradiol (E2), androgen to estrogen, and the metabolic ratios, and cortisol were significantly increased, indicating reproductive dysfunctions, while no differences were noted for males. The authors indicate that these effects in MEHP-exposed female zebrafishes may be closely associated with stress responses, such as elevated cortisol concentrations, indicating the assessed biomarkers, including cortisol, as adequate response biomarkers to evaluate the reproductive toxicity of endocrine disruptors that can serve as a methodological alternative to chronic toxicity testing. Another assessment verified the involvement of another phthalate, dibutyl phthalate (DBP), on male reproductive toxicity in the freshwater fish orange chromide (*Pseudetroplus maculatus*, [90]), where testicular toxicity was noted in the form of increased alkali-labile phosphates and total protein in plasma and testis, alongside testicular steroidogenic enzyme impairment and decreased follicle stimulating hormone, luteinizing hormone, and thyroid stimulating hormone and increased cortisol in serum concentrations, suggesting reproductive dysfunction and stress responses of the fish.

Regarding PCBs, most studies have exposed fish to commercial PCB mixtures such as Aroclor 1248 and 1254, which were mainly manufactured by Monsanto Chemical Corporation in the United States from 1930 to 1977 [91]. For example, Lerner et al. [92] reported decreased plasma thyroid hormone, triiodothyronine (T3), and cortisol concentrations in Atlantic salmon (*Salmo salar*) juveniles exposed to >1 μg·L^−1^ Aroclor 1254. Aluru et al. [93] and Vijayan et al. [94] exposed Atlantic charr (*Salvelinjus alpinus*) adults to >1 mg·kg^−1^ body mass of the same mixture, reporting impaired cortisol production and regulation in interrenal tissue, which they postulate could lead to stress-adaptation deficiencies. In another assessment, Iwanowicz et al. [15] exposed brown bullhead (*Ameiurus nebulosus*) adults intraperitoneally to 50 μg–5 mg·kg^−1^ body weight of Aroclor 1248. These authors reported decreased cortisol and T3 levels in plasma, as well as other effects, including decreased bactericidal activity and antibody production following exposure to pathogenic bacteria, leading to lower survival rates. Miller et al. [95] applied an in vitro mechanistic-approach assessment where isolated lake trout (*Salvelinus namaycush*) thymocytes cultures were treated with Aroclor 1254 in the presence and absence of cortisol or lipopolysaccharides. Cortisol was found to enhance the toxicity of both environmental contaminants, and the authors postulate that stressors that lead to increased cortisol production, but not lipopolysaccharide directly, may increase pollutant toxicity to lake trout thymocytes. In one of the few single PCB congener studies available, Stouthart et al. [96] demonstrated that PCB 126 alone can also affect stress responses in fish, as carp embryo exposure from 3.26 to 326 ng·L^−1^ altered whole-body ACTH and cortisol concentrations. However, these concentrations declined over time to below those found in controls.

Concerning pesticides, one study intraperitoneally exposed rainbow trout (*Oncorhynchus mykiss*) juveniles to 5, 20, or 50 mg·kg^−1^ body weight to the organochlorine endosulfan [97]. The authors reported decreased liver glycogen reserves and plasma cortisol concentrations, while two in vitro assessments conducted in head kidney cell cultures obtained from the same species following exposure to 18–366 μmol·L^−1^ endosulfan reported disruption of ACTH stimulated cortisol secretion [98,99].

Regarding PAH, acute and chronic stress responses were quantified in plasma cortisol concentrations in rainbow trout intraperitoneally administered naphthalene, β-naphthoflavone or benzo(a)pyrene at 10 mg·kg^−1^ [100]. Increased plasma cortisol concentrations were reported and increased even further with increased naphthalene and β-naphthoflavone treatments after acute stress. Interestingly, PAH-induced exposure to monoaminergic stress systems, with the dopaminergic system the most affected following acute stress, and the serotonergic system following chronic stress, indicated by increased concentrations of several metabolites in the brain. These metabolites also seemed to parallel PAH plasma cortisol effects. Thus, the authors suggested that plasma cortisol effects following PAH exposure could be partly mediated by monoaminergic system alterations.

In a similar study, Tintos et al. [101] intraperitoneally injected immature female rainbow trout with β-naphthoflavone or BaP benzo(a)pyrene at 10 mg·kg^−1^. The authors also reported increased plasma cortisol concentrations after 24 and 72 h of treatment with both PAHs, while no changes were noticed for 17β-estradiol concentrations. Several metabolic changes, such as increased plasma glucose and lactate concentrations and increased hepatic glycogenolysis, gluconeogenesis, and amino acid catabolism, were postulated as being directly associated with increased plasma cortisol concentrations.

Several studies exposing different fish species to water-soluble oil fractions are also found in the literature, as oil usually contains high amounts of PAHs. For example, Thomas and Rice [102] exposed juvenile coho salmon (*Oncorhynchus kisutch*) to the water-soluble fraction (WSF) of Cook Inlet crude oil at 75% of the LC50 (comprising the concentration that kills 50% of the study population). The study reported that exposed fish exhibited decreased swimming performance, increased plasma cortisol concentrations, and higher plasma cortisol concentrations when exposed to both WSF and forced swimming in a stamina tunnel. In another assessment, Kennedy and Farrell [103] exposed juvenile Pacific herring (*Clupea pallasi*) to three WSF concentrations of North Slope crude oil, both acutely (96 h) and chronically (9 weeks). Mean total PAH concentrations declined with time, and the WSF composition shifted toward larger and more substituted PAHs. Exposed fish displayed significant induction of hepatic cytochrome P450 content, ethoxyresorufin O-deethylase, and glutathione-S-transferase activities, indicating hydrocarbon bioavailability. Significant increases in plasma cortisol, lactate, and glucose were noted in acutely exposed fish, with concentrations returning to baseline levels after 96 h. Chronic exposure resulted in reduced cortisol responses and fewer significant plasma lactate and glucose concentration increases. Plasma Cl^−^, Na^+^, and K^+^ concentrations were significantly higher in the group exposed to 100 μg L^−1^ WSF by 96 h compared to control fish. Thus, the authors concluded that at least two important physiological herring systems are affected by chronic exposure to WSF, namely the ability to maintain ion homeostasis and organismal stress response.

## 7. Conclusions and Future Remarks

Fish, like other vertebrates, have developed several stress response mechanisms to cope with different stressors, including predation, intra and interspecific competition for resources, changing environmental conditions, and anthropogenic activities. These responses mainly generate the production of the cortisol hormone in fish, the primary glucocorticoid in this group. A shift towards non-invasive methods (e.g., employing scales, feces, mucus, and surrounding water) to determine this hormone is highlighted herein to the detriment of matrices obtained by invasive methods (e.g., whole body, eggs, fins, and plasma). The main analytical techniques currently used to quantify cortisol and its metabolites in fish comprise immunoassays, which have undergone a shift from RIAs to ELISAs, and LC-MS/MS. The latter represents a more sensitive and specific approach when compared to immunoassays, not affected by cross-activity and allowing for simultaneous quantification of multiple hormones. The matrices and techniques discussed herein can provide valuable information on fish stress responses and their ability to cope with environmental stressors, including exposure to organic pollutants (e.g., PAHs, PCBs, PFAS, and phthalates). The overall findings of the discussed studies indicate increased stress responses and other adverse effects in fish when exposed to these pollutants. Thus, it is crucial to identify key stressors affecting fish populations, such as organic pollutants, and monitor their stress responses, as these stressors can significantly affect the overall health of individuals and populations. The assessment of organic pollutants in fish can also provide insights into the magnitude and sources of local environmental contamination, allowing for the development of effective management and mitigation strategies that will safeguard the health and sustainability of aquatic ecosystems and exposed species.

Future research should focus on expanding the understanding of the mechanisms by which organic pollutants induce stress responses in fish. This knowledge will aid in the development of more effective mitigation strategies and the implementation of proper management practices to reduce the impact of these stressors. Additionally, investigating the long-term effects of chronic exposure to organic pollutants and their potential to disrupt fish populations’ reproductive success and genetic diversity should be a priority. Furthermore, the integration of molecular techniques, such as genomics and transcriptomics, with the assessment of stress responses in fish may provide a deeper understanding of the molecular pathways involved in stress responses, including the ones associated with pollutants. Future research should also focus on simultaneously evaluating different biomarkers to better understand how different physiological systems interact and respond to stressors. Ultimately, interdisciplinary efforts will be beneficial in unraveling the complexities of physiological responses in fish and developing comprehensive frameworks to address the challenges posed by stressors such as organic pollutants.

The study of fish stress responses and the assessment of organic pollutants in aquatic environments are vital for the health and sustainability of fish populations and aquatic ecosystems. By adopting non-invasive methods, utilizing advanced analytical techniques, and incorporating multidisciplinary approaches, researchers can continue to make significant strides in this field, ultimately contributing to the preservation of aquatic resources.

## Figures and Tables

**Table 1:** Advantages and disadvantages of different fish matrices employed in cortisol quantification.

Matrix	Invasiveness	Advantages	Disadvantages
Whole body	Invasive	High accuracy Reflects individual response levels Reflects plasma concentrations Can measure both basal and stress levels	Lethal Repeated measurements of the same individual are not possible
Eggs	Invasive	High accuracy Reflects individual response levels, when possible Low inter-sample variability	Lethal Repeated measurements of the same individual are not possible Not at the individual level when small eggs are pooled
Fins	Invasive	High accuracy Reflects individual response levels Can measure both basal and stress levels	Contact is required, which could lead to stress
Plasma	Invasive	High accuracy Reflects individual response levels Can measure both basal and stress levels Low inter-sample variability	Contact is required, which could lead to stress Can stress surrounding fish
Scales	Minimally invasive	Relatively non-invasive Indicates chronic stress Reflects individual response levels	Contact is required, which could lead to stress Difficult to associate chronic stress with stimulus timing
Feces	Non-invasive	Non-invasive Reflects individual response levels Reflects plasma concentrations Low inter-sample variability	Relatively higher sample sizes are required Lag time between the stimulus and excretion
Mucus	Non-invasive	Non-invasive Reflects plasma concentrations Well associated with stimulus timing	Contact is required, which could lead to stress High inter-sample variability
Water	Non-invasive	Non-invasive High accuracy Reflects plasma concentrations Moderate variability between samples	Difficult to assess individual levels Measurements at the individual level require fish isolation, which could lead to stress and the need for multiple tanks Sample can be lost by fish re-uptake and by degradation and surface absorption Lag time between the stimulus and excretion Higher volume of sample is needed, and longer extraction time than for other matrices

**Table 2:** Advantages and disadvantages of analytical cortisol quantification methods.

Method	Advantages	Disadvantages
Immunoassays	High sensitivities (as low as in the pg·mL^−1^ range) Use of small plasma or serum volumes Faster and cheaper compared to LC-MS/MS when measuring a single hormone No need to develop methods, as commercial assays for most hormones are already available	Require specific high-affinity antibodies, measuring a single hormone per assay The amplification technologies that give immunoassays high sensitivities are often less reliable than direct quantification methods Antibody cross-reactivity Limited dynamic range Matrix interferences Lack of internal standards to calculate recovery limits Reliance on external calibration
LC-MS/MS	Provides a robust platform with sufficient sensitivity and specificity for steroid hormone determination Able to determine cortisol in samples collected through non-invasive methods No cross-reactivity Can be used in the analytical validation of ELISAs and RIAs Allows for the simultaneous determination of several hormones Use of small sample volumes Fast analysis time	Detection of several hormones in a low volume of serum or plasma can be challenging Similarity of chemical structures and fragmentation patterns demands sufficient chromatographic separation, especially for isomers High instrument costs Greater technical complexity, speed, and turnaround of analysis Greater complexity in sample preparation is required to avoid blockages and sample matrix effects
GC-MS	Fast and precise Excellent chromatographic resolution Multi-class profiling potential Intensive fragmentation patterns leading to better structure elucidation	More labor-intensive, requiring derivatization Reproducibility issues due to incomplete compound derivatization

## Data Availability

The authors confirm that the data supporting the findings of this study are available within the article and its references.

## References

[1] Balasch J.C., Tort L. (2019). Netting the Stress Responses in Fish. Front. Endocrinol..

[2] Buschmann J. (2013). The OECD Guidelines for the Testing of Chemicals and Pesticides. Fish Biol..

[3] Schreck C.B., Tort L. (2016). The Concept of Stress in Fish. Fish Physiology.

[4] Hook S.E., Gallagher E.P., Batley G.E. (2014). The Role of Biomarkers in the Assessment of Aquatic Ecosystem Health. Integr. Environ. Assess. Manag..

[5] Smit M.G.D., Bechmann R.K., Hendriks A.J., Skadsheim A., Larsen B.K., Baussant T., Bamber S., Sanni S. (2009). Relating Biomarkers to Whole-Organism Effects Using Species Sensitivity Distributions: A Pilot Study for Marine Species Exposed to Oil. Environ. Toxicol. Chem..

[6] de la Espino G.L., Pulido S.H., Pérez J.L.C. (2011). Organismos Indicadores de la Calidad del Agua y de la Contaminación (Bioindicadores).

[7] Holt E.A., Miller S.W. (2011). Bioindicators: Using Organisms to Measure Environmental Impacts. Nat. Educ. Knowl..

[8] Lemos L., Gantiva L., Kaylor C., Sanchez A., Quinete N. (2022). American Oysters as Bioindicators of Emerging Organic Contaminants in Florida, United States. Sci. Total Environ..

[9] Quinete N., Wu Q., Zhang T., Yun S.H., Moreira I., Kannan K. (2009). Specific Profiles of Perfluorinated Compounds in Surface and Drinking Waters and Accumulation in Mussels, Fish, and Dolphins from Southeastern Brazil. Chemosphere.

[10] Wendelaar Bonga S.E. (1997). The Stress Response in Fish. Physiol. Rev..

[11] Tejchman K., Kotfis K., Sieńko J. (2021). Biomarkers and Mechanisms of Oxidative Stress—Last 20 Years of Research with an Emphasis on Kidney Damage and Renal Transplantation. Int. J. Mol. Sci..

[12] Barton B.A. (2002). Stress in Fishes: A Diversity of Responses with Particular Reference to Changes in Circulating Corticosteroids. Integr. Comp. Biol..

[13] Zimmer K.E., Montaño M., Olsaker I., Dahl E., Berg V., Karlsson C., Murk A.J., Skaare J.U., Ropstad E., Verhaegen S. (2011). In Vitro Steroidogenic Effects of Mixtures of Persistent Organic Pollutants (POPs) Extracted from Burbot (*Lota Lota*) Caught in Two Norwegian Lakes. Sci. Total Environ..

[14] Sadoul B., Geffroy B. (2019). Measuring Cortisol, the Major Stress Hormone in Fishes. J. Fish Biol..

[15] Iwanowicz L.R., Blazer V.S., McCormick S.D., VanVeld P.A., Ottinger C.A. (2009). Aroclor 1248 Exposure Leads to Immunomodulation, Decreased Disease Resistance and Endocrine Disruption in the Brown Bullhead, *Ameiurus Nebulosus*. Aquat. Toxicol..

[16] Laflamme J.-S., Couillard Y., Campbell P.G., Hontela A. (2000). Interrenal Metallothionein and Cortisol Secretion in Relation to Cd, Cu, and Zn Exposure in Yellow Perch, Perca Flavescens, from Abitibi Lakes. Can. J. Fish. Aquat. Sci..

[17] Robertson L., Thomas P., Arnold C.R., Trant J.M. (1987). Plasma Cortisol and Secondary Stress Responses of Red Drum to Handling, Transport, Rearing Density, and a Disease Outbreak. Progress. Fish-Culturist.

[18] Alfonso S., Houdelet C., Bessa E., Geffroy B., Sadoul B. (2023). Water Temperature Explains Part of the Variation in Basal Plasma Cortisol Level within and between Fish Species. J. Fish Biol..

[19] Kitagawa A.T., Costa L.S., Paulino R.R., Luz R.K., Rosa P.V., Guerra-Santos B., Fortes-Silva R. (2015). Feeding Behavior and the Effect of Photoperiod on the Performance and Hematological Parameters of the Pacamã Catfish (*Lophiosilurus alexandri*). Appl. Anim. Behav. Sci..

[20] Pavlidis M., Greenwood L., Paalavuo M., Mölsä H., Laitinen J.T. (1999). The Effect of Photoperiod on Diel Rhythms in Serum Melatonin, Cortisol, Glucose, and Electrolytes in the Common Dentex, Dentex Dentex. Gen. Comp. Endocrinol..

[21] Sánchez-Vázquez F.J., López-Olmeda J.F., Vera L.M., Migaud H., López-Patiño M.A., Míguez J.M. (2019). Environmental Cycles, Melatonin, and Circadian Control of Stress Response in Fish. Front. Endocrinol..

[22] Strange R.J. (1980). Acclimation Temperature Influences Cortisol and Glucose Concentrations in Stressed Channel Catfish. Trans. Am. Fish. Soc..

[23] Hwang I.J., Kim S.Y., Kim H.B., Baek H.J. (2012). Changes in Plasma Sex Steroid and Cortisol Levels during Annual Reproductive Cycle of Ribbed Gunnel, *Dictyosoma burgeri*. Dev. Reprod..

[24] Pottinger T.G., Balm P.H.M., Pickering A.D. (1995). Sexual Maturity Modifies the Responsiveness of the Pituitary-Interrenal Axis to Stress in Male Rainbow Trout. Gen. Comp. Endocrinol..

[25] Cyr N.E., Romano L.M. (2009). Identifying Hormonal Habituation in Field Studies of Stress. Gen. Comp. Endocrinol..

[26] Mommsen T.P., Vijayan M.M., Moon T.W. (1999). Cortisol in Teleosts: Dynamics, Mechanisms of Action, and Metabolic Regulation. Rev. Fish Biol. Fish..

[27] Spiga F., Waite E.J., Liu Y., Kershaw Y.M., Aguilera G., Lightman S.L. (2011). ACTH-Dependent Ultradian Rhythm of Corticosterone Secretion. Endocrinology.

[28] Davis K.B., McEntire M. (2009). Comparison of the Cortisol and Glucose Stress Response to Acute Confinement among White Bass, *Morone chrysops*, Striped Bass, *Morone saxatilis*, and Sunshine Bass, *Morone chrysops* × *Morone saxatilis*. J. World Aquac. Soc..

[29] Martínez-Porchas M., Martínez-Córdova L.R., Ramos-Enriquez R. (2009). Cortisol and Glucose: Reliable Indicators of Fish Stress?. Panam. J. Aquat. Sci..

[30] Faught E., Aluru N., Vijayan M.M. (2016). The Molecular Stress Response. Fish Physiol..

[31] Das C., Thraya M., Vijayan M.M. (2018). Nongenomic Cortisol Signaling in Fish. Gen. Comp. Endocrinol..

[32] Scott A.P., Ellis T. (2007). Measurement of Fish Steroids in Water—A Review. Gen. Comp. Endocrinol..

[33] Pickering A.D., Pottinger T.G. (1989). Stress Responses and Disease Resistance in Salmonid Fish: Effects of Chronic Elevation of Plasma Cortisol. Fish Physiol. Biochem..

[34] Barry T.P., Malison J.A., Held J.A., Parrish J.J. (1995). Ontogeny of the Cortisol Stress Response in Larval Rainbow Trout. Gen. Comp. Endocrinol..

[35] Stratholt M.L., Donaldson E.M., Liley N.R. (1997). Stress Induced Elevation of Plasma Cortisol in Adult Female Coho Salmon (*Oncorhynchus kisutch*), Is Reflected in Egg Cortisol Content, but Does Not Appear to Affect Early Development. Aquaculture.

[36] Tort L., Kargacin B., Torres P., Giralt M., Hidalgo J. (1996). The Effect of Cadmium Exposure and Stress on Plasma Cortisol, Metallothionein Levels and Oxidative Status in Rainbow Trout (*Oncorhynchus mykiss*) Liver. Comp. Biochem. Physiol. Part C Pharmacol. Toxicol. Endocrinol..

[37] Ellis T., Bagwell N., Pond M., Baynes S., Scott A.P. (2007). Acute Viral and Bacterial Infections Elevate Water Cortisol Concentrations in Fish Tanks. Aquaculture.

[38] Laberge F., Yin-Liao I., Bernier N.J. (2019). Temporal Profiles of Cortisol Accumulation and Clearance Support Scale Cortisol Content as an Indicator of Chronic Stress in Fish. Conserv. Physiol..

[39] Ding J., Finstad B., Gansel L.C., Tveten A.-K., Blindheim S.H., Cao Y. (2023). Comparative Assessment of Plasma Cortisol and Fecal Corticoid Metabolites (FCM) of Atlantic Salmon (*Salmo salar* L.) Subjected to Acute- and Long-Term Stress. Aquaculture.

[40] Murtagh R., Behringer V., Deschner T. (2013). LC-MS as a Method for Non-Invasive Measurement of Steroid Hormones and Their Metabolites in Urine and Faeces of Animals. Wien. Tierärztliche Mon. Vet. Med. Austria.

[41] McDonald M.D., Wood C.M. (2004). The Effect of Chronic Cortisol Elevation on Urea Metabolism and Excretion in the Rainbow Trout (*Oncorhynchus mykiss*). J. Comp. Physiol. B Biochem. Syst. Environ. Physiol..

[42] Meling V.A., Berge K., Knudsen D.L., Rønning P.O., Brede C. (2022). Monitoring Farmed Fish Welfare by Measurement of Cortisol as a Stress Marker in Fish Feces by Liquid Chromatography Coupled with Tandem Mass Spectrometry. Molecules.

[43] Uren Webster T.M., Rodriguez-Barreto D., Consuegra S., Garcia de Leaniz C. (2020). Cortisol-Related Signatures of Stress in the Fish Microbiome. Front. Microbiol..

[44] Samaras A., Pavlidis M. (2022). Fish Scales Produce Cortisol upon Stimulation with ACTH. Animals.

[45] Boulton K., Couto E., Grimmer A.J., Earley R.L., Canario A.V.M., Wilson A.J., Walling C.A. (2015). How Integrated Are Behavioral and Endocrine Stress Response Traits? A Repeated Measures Approach to Testing the Stress-Coping Style Model. Ecol. Evol..

[46] Zuberi A., Brown C., Ali S. (2014). Effect of Confinement on Waterborne and Whole Body Cortisol in Wild and Captive-Eared Rainbowfish (*Melanoteania duboulayi*). Int. J. Agric. Biol..

[47] Fanouraki E., Papandroulakis N., Ellis T., Mylonas C.C., Scott A.P., Pavlidis M. (2008). Water Cortisol Is a Reliable Indicator of Stress in European Sea Bass, *Dicentrarchus labrax*. Behaviour.

[48] Wong S.C., Dykstra M., Campbell J.M., Earley R.L. (2008). Measuring Water-Borne Cortisol in Convict Cichlids (*Amatitlania nigrofasciata*): Is the Procedure a Stressor?. Behaviour.

[49] Palme R. (2005). Measuring Fecal Steroids: Guidelines for Practical Application. Ann. N. Y. Acad. Sci..

[50] Lemos L.S., Olsen A., Smith A., Chandler T., Larson S., Hunt K., Torres L. (2020). Assessment of Fecal Steroid and Thyroid Hormone Metabolites in Eastern North Pacific Gray Whales. Conserv. Physiol..

[51] Millspaugh J.J., Washburn B.E. (2004). Use of Fecal Glucocorticoid Metabolite Measures in Conservation Biology Research: Considerations for Application and Interpretation. Gen. Comp. Endocrinol..

[52] Ellis T., Sanders M.B., Scott A.P. (2013). Non-Invasive Monitoring of Steroids in Fishes. Vet. Med. Austria.

[53] Simontacchi C., Poltronieri C., Carraro C., Bertotto D., Xiccato G., Trocino A., Radaelli G. (2008). Alternative Stress Indicators in Sea Bass *Dicentrarchus labrax* L. J. Fish Biol..

[54] Bertotto D., Poltronieri C., Negrato E., Majolini D., Radaelli G., Simontacchi C. (2010). Alternative Matrices for Cortisol Measurement in Fish. Aquac. Res..

[55] Gozdowska M., Sokołowska E., Pomianowski K., Kulczykowska E. (2022). Melatonin and Cortisol as Components of the Cutaneous Stress Response System in Fish: Response to Oxidative Stress. Comp. Biochem. Physiol. Part A Mol. Integr. Physiol..

[56] Guardiola F.A., Cuesta A., Esteban M.Á. (2016). Using Skin Mucus to Evaluate Stress in Gilthead Seabream (*Sparus aurata* L.). Fish Shellfish Immunol..

[57] Carbajal A., Reyes-López F.E., Tallo-Parra O., Lopez-Bejar M., Tort L. (2019). Comparative Assessment of Cortisol in Plasma, Skin Mucus and Scales as a Measure of the Hypothalamic-Pituitary-Interrenal Axis Activity in Fish. Aquaculture.

[58] Auperin B., Geslin M. (2008). Plasma Cortisol Response to Stress in Juvenile Rainbow Trout Is Influenced by Their Life History during Early Development and by Egg Cortisol Content. Gen. Comp. Endocrinol..

[59] Barry T.P., Lapp A.F., Kayes T.B., Malison J.A. (1993). Validation of a Microtitre Plate ELISA for Measuring Cortisol in Fish and Comparison of Stress Responses of Rainbow Trout (*Oncorhynchus mykiss*) and Lake Trout (*Salvelinus namaycush*). Aquaculture.

[60] de Pablo F., Scanes C.G., Weintraub B.D. (1993). Handbook of Endocrine Research Techniques.

[61] Aydin S. (2015). A Short History, Principles, and Types of ELISA, and Our Laboratory Experience with Peptide/Protein Analyses Using ELISA. Peptides.

[62] Weltring A., Schaebs F.S., Perry S.E., Deschner T. (2012). Simultaneous Measurement of Endogenous Steroid Hormones and Their Metabolites with LC–MS/MS in Faeces of a New World Primate Species, *Cebus capucinus*. Physiol. Behav..

[63] Boggs A.S.P., Bowden J.A., Galligan T.M., Guillette L.J., Kucklick J.R. (2016). Development of a Multi-Class Steroid Hormone Screening Method Using Liquid Chromatography/Tandem Mass Spectrometry (LC-MS/MS). Anal. Bioanal. Chem..

[64] Keevil B.G. (2016). LC–MS/MS Analysis of Steroids in the Clinical Laboratory. Clin. Biochem..

[65] Taylor A.E., Keevil B., Huhtaniemi I.T. (2015). Mass Spectrometry and Immunoassay: How to Measure Steroid Hormones Today and Tomorrow. Eur. J. Endocrinol..

[66] Keevil B.G. (2013). Novel Liquid Chromatography Tandem Mass Spectrometry (LC-MS/MS) Methods for Measuring Steroids. Best Pract. Res. Clin. Endocrinol. Metab..

[67] Nouri M.-Z., Kroll K.J., Webb M., Denslow N.D. (2020). Quantification of Steroid Hormones in Low Volume Plasma and Tissue Homogenates of Fish Using LC-MS/MS. Gen. Comp. Endocrinol..

[68] Maciążek-Jurczyk M., Bessonneau V., Ings J., Bragg L., McMaster M., Servos M.R., Bojko B., Pawliszyn J. (2020). Development of a Thin-Film Solid-Phase Microextraction (TF-SPME) Method Coupled to Liquid Chromatography and Tandem Mass Spectrometry for High-Throughput Determination of Steroid Hormones in White Sucker Fish Plasma. Anal. Bioanal. Chem..

[69] Palmer K., Bangma J.T., Reiner J.L., Bonde R.K., Korte J.E., Boggs A.S.P., Bowden J.A. (2019). Per- and Polyfluoroalkyl Substances (PFAS) in Plasma of the West Indian Manatee (*Trichechus manatus*). Mar. Pollut. Bull..

[70] EPA Method 1698: Steroids and Hormones in Water, Soil, Sediment, and Biosolids by HRGC/HRMS. https://www.epa.gov/sites/default/files/2015-10/documents/method_1698_2007.pdf.

[71] Rambaud L., Monteau F., Deceuninck Y., Bichon E., André F., Le Bizec B. (2007). Development and Validation of a Multi-Residue Method for the Detection of a Wide Range of Hormonal Anabolic Compounds in Hair Using Gas Chromatography–Tandem Mass Spectrometry. Anal. Chim. Acta.

[72] Bowden J.A., Colosi D.M., Mora-Montero D.C., Garrett T.J., Yost R.A. (2009). Enhancement of Chemical Derivatization of Steroids by Gas Chromatography/Mass Spectrometry (GC/MS). J. Chromatogr. B.

[73] Gluth G., Hanke W. (1985). A Comparison of Physiological Changes in Carp, *Cyprinus carpio*, Induced by Several Pollutants at Sublethal Concentrations. Ecotoxicol. Environ. Saf..

[74] Hontela A., Rasmussen J., Audet C., Chevalier G. (1992). Impaired Cortisol Stress Response in Fish from Environments Polluted by PAHs, PCBs, and Mercury. Arch. Environ. Contam. Toxicol..

[75] Schumann S., Negrato E., Piva E., Pietropoli E., Bonato M., Irato P., Marion A., Santovito G., Bertotto D. (2022). Species-Specific Physiological Responses in Freshwater Fish Exposed to Anthropogenic Perfluorochemical (Pfas) Pollution. SSRN Electron. J..

[76] Rohonczy J., O’Dwyer K., Rochette A., Robinson S.A., Forbes M.R. (2021). Meta-Analysis Shows Environmental Contaminants Elevate Cortisol Levels in Teleost Fish—Effect Sizes Depend on Contaminant Class and Duration of Experimental Exposure. Sci. Total Environ..

[77] Celino-Brady F.T., Lerner D.T., Seale A.P. (2021). Experimental Approaches for Characterizing the Endocrine-Disrupting Effects of Environmental Chemicals in Fish. Front. Endocrinol..

[78] Grosell M., Pasparakis C. (2021). Physiological Responses of Fish to Oil Spills. Ann. Rev. Mar. Sci..

[79] Bojarski B., Witeska M. (2020). Blood Biomarkers of Herbicide, Insecticide, and Fungicide Toxicity to Fish—A Review. Environ. Sci. Pollut. Res..

[80] Bury N.R., Jie L., Flik G., Lock R.A.C., Bonga S.E.W. (1998). Cortisol Protects against Copper Induced Necrosis and Promotes Apoptosis in Fish Gill Chloride Cells in Vitro. Aquat. Toxicol..

[81] De Boeck G., De Wachter B., Vlaeminck A., Blust R. (2003). Effect of Cortisol Treatment and/or Sublethal Copper Exposure on Copper Uptake and Heat Shock Protein Levels in Common Carp, *Cyprinus carpio*. Environ. Toxicol. Chem..

[82] Hontela A., Daniel C., Rasmussen J.B. (1997). Structural and Functional Impairment of the Hypothalamo-Pituitary-Interrenal Axis in Fish Exposed to Bleached Kraft Mill Effluent in the St Maurice River, Quebec. Ecotoxicology.

[83] Marentette J.R., Tong S., Balshine S. (2013). The Cortisol Stress Response in Male Round Goby (*Neogobius melanostomus*): Effects of Living in Polluted Environments?. Environ. Biol. Fishes.

[84] Johnson L.L., Anulacion B.F., Arkoosh M.R., Burrows D.G., da Silva D.A.M., Dietrich J.P., Myers M.S., Spromberg J., Ylitalo G.M., Tierney K.B., Farrell A.P., Brauner C.J. (2013). Effects of Legacy Persistent Organic Pollutants (POPs) in Fish—Current and Future Challenges. Fish Physiology.

[85] Dietz R., Letcher R.J., Desforges J.-P., Eulaers I., Sonne C., Wilson S., Andersen-Ranberg E., Basu N., Barst B.D., Bustnes J.O. (2019). Current State of Knowledge on Biological Effects from Contaminants on Arctic Wildlife and Fish. Sci. Total Environ..

[86] Gandar A., Laffaille P., Canlet C., Tremblay-Franco M., Gautier R., Perrault A., Gress L., Mormède P., Tapie N., Budzinski H. (2017). Adaptive Response under Multiple Stress Exposure in Fish: From the Molecular to Individual Level. Chemosphere.

[87] Pottinger T.G. (2017). Modulation of the Stress Response in Wild Fish Is Associated with Variation in Dissolved Nitrate and Nitrite. Environ. Pollut..

[88] Mortensen A.S., Letcher R.J., Cangialosi M.V., Chu S., Arukwe A. (2011). Tissue Bioaccumulation Patterns, Xenobiotic Biotransformation and Steroid Hormone Levels in Atlantic Salmon (*Salmo salar*) Fed a Diet Containing Perfluoroactane Sulfonic or Perfluorooctane Carboxylic Acids. Chemosphere.

[89] Park C.-B., Kim G.-E., Kim Y.J., On J., Park C.-G., Kwon Y.-S., Pyo H., Yeom D.-H., Cho S.-H. (2020). Reproductive Dysfunction Linked to Alteration of Endocrine Activities in Zebrafish Exposed to Mono-(2-Ethylhexyl) Phthalate (MEHP). Environ. Pollut..

[90] Sruthi M., Raibeemol K.P., Chitra K.C. (2021). Involvement of Dibutyl Phthalate on Male Reproductive Toxicity in the Freshwater Fish *Pseudetroplus maculatus* (Bloch, 1795). J. Appl. Aquac..

[91] ATSDR Toxicological Profile for Polychlorinated Biphenyls (PCBs). https://www.atsdr.cdc.gov/toxprofiles/tp17.pdf.

[92] Lerner D., Bjornsson B., McCormick S. (2007). Effects of Aqueous Exposure to Polychlorinated Biphenyls (Aroclor 1254) on Physiology and Behavior of Smolt Development of Atlantic Salmon. Aquat. Toxicol..

[93] Aluru N., Jorgensen E.H., Maule A.G., Vijayan M.M. (2004). PCB Disruption of the Hypothalamus-Pituitary-Interrenal Axis Involves Brain Glucocorticoid Receptor Downregulation in Anadromous Arctic Charr. Am. J. Physiol. Integr. Comp. Physiol..

[94] Vijayan M.M., Aluru N., Maule A.G., Jørgensen E.H. (2006). Fasting Augments PCB Impact on Liver Metabolism in Anadromous Arctic Char. Toxicol. Sci..

[95] Miller G.G., Sweet L.I., Adams J.V., Omann G.M., Passino-Reader D.R., Meier P.G. (2002). In Vitro Toxicity and Interactions of Environmental Contaminants (Arochlor 1254 and Mercury) and Immunomodulatory Agents (Lipopolysaccharide and Cortisol) on Thymocytes from Lake Trout (*Salvelinus namaycush*). Fish Shellfish Immunol..

[96] Stouthart X.J.H.X., Huijbregts M.A.J., Balm P.H.M., Lock R.A.C., Bonga S.E.W. (1998). Endocrine Stress Response and Abnormal Development in Carp (*Cyprinus carpio*) Larvae after Exposure of the Embryos to PCB 126. Fish Physiol. Biochem..

[97] Benguira S., Leblond V.S., Weber J.P., Hontela A. (2002). Loss of Capacity to Elevate Plasma Cortisol in Rainbow Trout (*Oncorhynchus mykiss*) Treated with a Single Injection of o,p′-Dichlorodiphenyldichloroethane. Env. Toxicol Chem.

[98] Dorval J., Leblond V.S., Hontela A. (2003). Oxidative Stress and Loss of Cortisol Secretion in Adrenocortical Cells of Rainbow Trout (*Oncorhynchus mykiss*) Exposed in Vitro to Endosulfan, an Organochlorine Pesticide. Aquat. Toxicol..

[99] Lacroix M., Hontela A. (2003). The Organochlorine o,p′-DDD Disrupts the Adrenal Steroidogenic Signaling Pathway in Rainbow Trout (*Oncorhynchus mykiss*). Toxicol. Appl. Pharmacol..

[100] Gesto M., Soengas J.L., Míguez J.M. (2008). Acute and Prolonged Stress Responses of Brain Monoaminergic Activity and Plasma Cortisol Levels in Rainbow Trout Are Modified by PAHs (Naphthalene, β-Naphthoflavone and Benzo(a)Pyrene) Treatment. Aquat. Toxicol..

[101] Tintos A., Gesto M., Míguez J.M., Soengas J.L. (2008). β-Naphthoflavone and Benzo(a)Pyrene Treatment Affect Liver Intermediary Metabolism and Plasma Cortisol Levels in Rainbow Trout *Oncorhynchus mykiss*. Ecotoxicol. Environ. Saf..

[102] Thomas R.E., Rice S.D. (1987). Effect of Water-Soluble Fraction of Cook Inlet Crude Oil on Swimming Performance and Plasma Cortisol in Juvenile Coho Salmon (*Oncorhynchus kisutch*). Comp. Biochem. Physiol. Part C Comp. Pharmacol..

[103] Kennedy C.J., Farrell A.P. (2005). Ion Homeostasis and Interrenal Stress Responses in Juvenile Pacific Herring, *Clupea Pallasi*, Exposed to the Water-Soluble Fraction of Crude Oil. J. Exp. Mar. Bio. Ecol..

